# Traffic-Related Air Pollution and Congenital Anomalies in Barcelona

**DOI:** 10.1289/ehp.1306802

**Published:** 2014-01-03

**Authors:** Anna Schembari, Mark J. Nieuwenhuijsen, Joaquin Salvador, Audrey de Nazelle, Marta Cirach, Payam Dadvand, Rob Beelen, Gerard Hoek, Xavier Basagaña, Martine Vrijheid

**Affiliations:** 1 Centre for Research in Environmental Epidemiology (CREAL), Barcelona, Spain; 2 CIBER Epidemiologia y Salud Publica (CIBERESP), Barcelona, Spain; 3 Universitat Pompeu Fabra (UPF), Barcelona, Spain; 4 Servei de sistema d’Informació Sanitaria, Agencia de Salut Pública de Barcelona, Barcelona, Spain; 5 Centre for Environmental Policy, Imperial College London, London, United Kingdom; 6 Institute for Risk Assessment Sciences (IRAS), University of Utrecht, Utrecht, the Netherlands

## Abstract

Background*:* A recent meta-analysis suggested evidence for an effect of exposure to ambient air pollutants on risk of certain congenital heart defects. However, few studies have investigated the effects of traffic-related air pollutants with sufficient spatial accuracy.

Objectives: We estimated associations between congenital anomalies and exposure to traffic-related air pollution in Barcelona, Spain.

Method: Cases with nonchromosomal anomalies (*n* = 2,247) and controls (*n* = 2,991) were selected from the Barcelona congenital anomaly register during 1994–2006. Land use regression models from the European Study of Cohorts for Air Pollution Effects (ESCAPE), were applied to residential addresses at birth to estimate spatial exposure to nitrogen oxides and dioxide (NO_x_, NO_2_)_,_ particulate matter with diameter ≤ 10 μm (PM_10_), 10–2.5 μm (PM_coarse_), ≤ 2.5 μm (PM_2.5_), and PM_2.5_ absorbance. Spatial estimates were adjusted for temporal trends using data from routine monitoring stations for weeks 3–8 of each pregnancy. Logistic regression models were used to calculate odds ratios (ORs) for 18 congenital anomaly groups associated with an interquartile-range (IQR) increase in exposure estimates.

Results: In spatial and spatiotemporal exposure models, we estimated statistically significant associations between an IQR increase in NO_2_ (12.2 μg/m^3^) and coarctation of the aorta (OR_spatiotemporal_ = 1.15; 95% CI: 1.01, 1.31) and digestive system defects (OR_spatiotemporal_ = 1.11; 95% CI: 1.00, 1.23), and between an IQR increase in PM_coarse_ (3.6 μg/m^3^) and abdominal wall defects (OR_spatiotemporal_ = 1.93; 95% CI: 1.37, 2.73). Other statistically significant increased and decreased ORs were estimated based on the spatial model only or the spatiotemporal model only, but not both.

Conclusions: Our results overall do not indicate an association between traffic-related air pollution and most groups of congenital anomalies. Findings for coarctation of the aorta are consistent with those of the previous meta-analysis.

Citation: Schembari A, Nieuwenhuijsen MJ, Salvador J, de Nazelle A, Cirach M, Dadvand P, Beelen R, Hoek G, Basagaña X, Vrijheid M. 2014. Traffic-related air pollution and congenital anomalies in Barcelona. Environ Health Perspect 122:317–323; http://dx.doi.org/10.1289/ehp.1306802

## Introduction

There is a growing body of epidemiologic evidence suggesting that exposure to ambient air pollution may adversely affect the fetus and newborn. Recent studies have found associations between particulate matter (PM) and increased risk of low birth weight, preterm birth, and decrease in birth weight (Dadvand et al. 2013; Pedersen et al. 2013; Sapkota et al. 2010). Major congenital anomalies include structural defects such as heart defects and neural tube defects and chromosomal abnormalities such as Down syndrome, and are diagnosed in 2–4% of births [World Health Organization (WHO) 2012]. They are a main cause of infant mortality and important contributor to childhood and adult morbidity, but their etiology remains largely unknown (Dolk and Vrijheid 2003).

The evidence for an impact of ambient air pollution on congenital anomaly risk is still limited (Agay-Shay et al. 2013; Dadvand et al. 2011a, 2011b; Dolk et al. 2010; Gilboa et al. 2005; Hansen et al. 2009; Hwang and Jaakola 2008; Kim et al. 2007; Marshall et al. 2010; Padula et al. 2013a, 2013b; Rankin et al. 2009; Ritz et al. 2002; Strickland et al. 2009; Vrijheid et al. 2011). Previous studies have focused primarily on the routinely assessed pollutants; only four studies (Agay-Shay et al. 2013; Marshall et al. 2010; Padula et al. 2013a, 2013b) included other specific traffic-related air pollutants such as PM_2.5_ (PM with diameter ≤ 2.5 μm). Cardiac anomalies or oral clefts were most frequently studied, but available evidence on other anomaly groups, such as defects of the nervous, digestive, or respiratory systems, is scarce (Dolk et al. 2010; Padula et al. 2013a; Rankin et al. 2009). Summary estimates from a recent meta-analysis (Vrijheid et al. 2011) indicated that nitrogen dioxide (NO_2_) and sulfur dioxide (SO_2_) were associated with two congenital heart anomalies—coarctation of the aorta and tetralogy of Fallot—and that PM_10_ (PM with diameter ≤ 10 μm) was associated with atrial septal defects. Except for Dadvand et al. (2011b), exposure assessments in previous studies were based on monitoring data from a limited number of fixed-site stations, and thus did not account for the strong spatial intra-city variation that characterizes traffic-related air pollution (Cyrys et al. 2012; Eeftens et al. 2012b). Dadvand et al. (2011b) estimated spatiotemporal exposure to black smoke and SO_2_ with higher spatial resolution. The use of more precise spatial air pollution models in urban areas is increasingly recommended to reduce exposure misclassification (Hoek et al. 2008; Jerrett et al. 2004) in studies of traffic-related air pollution and adverse birth outcomes (Aguilera et al. 2009; Ballester et al. 2010; Brauer et al. 2008). Moreover, in studies of congenital anomalies, spatial models need to be combined with temporal adjustments to account for the very specific etiologically relevant time windows of exposure (Ritz and Wilhelm 2008).

Barcelona, Spain, is among the most polluted cities in Europe (Cyrys et al. 2012; Eeftens et al. 2012b). This is partly attributable to its geography; high traffic density (Ajuntament de Barecelona 2007), which is four times higher than London; and large proportion of diesel-powered vehicles, currently 50% (Reche et al. 2011). In Barcelona we developed a refined spatial air pollution exposure metric using a land use regression model (Beelen et al. 2013; Eeftens et al. 2012a). At the same time, Barcelona has a high-quality population-based congenital malformation register, which has collected detailed data on cases of congenital anomalies and control births for > 15 years (Greenlees et al. 2011). Using these two data sources, we investigated associations between specific groups of congenital anomalies and intra-urban traffic-related air pollution exposures during sensitive time windows in pregnancy.

## Methods

*Study population*. This study used a population-based case–control design. The source population consisted of congenital anomaly cases and control births from the Barcelona congenital anomalies register, Registro Defectios Congenitos Barcelona (REDCB), which has accrued approximately 13,000 births/year from nearly all maternity units in the city since its start in 1992. The REDCB is part of the Service of Health Information Systems (Servei de Sistemes d’Informació Sanitaria) in the Public Health Agency of Barcelona (Agència de Salut Pública de Barcelona) and a member of the European surveillance of congenital anomalies (EUROCAT) (Greenlees et al. 2011). For the REDCB, full-time nurses actively search for new cases at delivery units, pediatric departments, cytogenetic laboratories, pathology departments, prenatal diagnosis units, and pediatric cardiology services; cases of congenital anomalies suspected but not detected at birth are further followed up until age 1 year. Controls are continuously selected as a 2% representative sample of all live births in Barcelona hospitals, by random date of birth sampling, independent of the cases (nonmatched). Clinical information, including maternal age, is collected from hospital records. As part of the REDCB, information on smoking and education is collected by interview with the mother, using a standardized questionnaire (Salvador et al. 2005). Parental informed consent is completed before the interview, and all consenting cases detected during the maternal period at the hospital and the control mothers are interviewed (35% and 95%, respectively); interviews cannot be held with mothers of cases diagnosed or communicated to the registry authority after the maternity period. In these cases, smoking and maternal education information is abstracted from clinical history records. Further, for the purposes of the current study, the residential addresses of the cases and controls were linked to the MEDEA (Mortalidad en áreas pequeñas Españolas y Desigualdades Socioeconómicas y Ambientales) socioeconomic deprivation index (Dominguez-Berjon et al. 2008) at census-tract level.

The present study was approved by the Clinical Research Ethics Committee of PS–Mar (CEIC project 2008/3115/I and 2006/3394/I) and determined to be exempt from separate informed consent requirements.

*Definition of cases and controls*. Controls were all live births enrolled in the REDCB during 1994–2006 with a residential address at birth in the city of Barcelona. Of the 3,149 controls included in the REDCB during that time period, 158 were excluded because of a residential address that could not be geocoded or that was outside Barcelona, giving a total of 2,991 (95.0%) controls for analysis.

Cases were live births, stillbirths after 20 weeks of gestation, or terminations of pregnancy after prenatal diagnosis of congenital anomalies, enrolled in the REDCB and born or terminated between 1994 and 2006, with at least one major anomaly in the EUROCAT list of subgroups of congenital anomalies (EUROCAT 2009). Cases with only minor congenital anomalies, such as facial asymmetry (code Q67.0) or talipes or pes calcaneovalgus (Q66.4) were excluded from the study, following the EUROCAT exclusion list (EUROCAT 2009). The range of codes for inclusion was, according to the *International Classification of Diseases, 10th Revision* (ICD-10) (WHO 1993), the Q chapter. Cases with chromosomal anomalies (Q90–Q92, Q93, Q96–Q99; *n* = 719), and teratogenic and genetic syndromes (Q86, Q87; *n* = 72) were excluded. Twins were treated as one outcome (12 pairs) and classified as a case if one or both had a congenital anomaly; siblings were classified as separate outcomes. Cases with residential addresses that could not be geocoded or that were outside Barcelona were excluded (*n* = 166), as were spontaneous abortion before 20 weeks of gestation (*n* = 5). In addition, we excluded cases that could not be classified according to the timing of the vulnerable time window of exposure (i.e., 3–8 weeks gestation, when organogenesis occurs) because any information on birth date, gestational age (GA), and date of the last menstrual period (LMP) (*n* = 74) was available. Of 3,295 cases, 2,247 (68%) were included in analysis. Cases were classified into anomaly subgroups following EUROCAT guidelines (EUROCAT 2009). An *a priori* decision was made to analyze the following common subgroups of anomalies: neural tube defects (Q00, Q01, Q05), congenital heart disease (Q20–Q26), respiratory system defects (Q30–Q34), orofacial clefts (Q35–Q37), digestive system defects (Q38–Q38, Q402–Q409, Q41–Q45, Q790), abdominal wall defects (Q792, Q793, Q795), urinary system defects (Q60–Q64, Q794), hypospadias (Q54), and limb reduction defects (Q650–652, Q658–Q659, Q660, Q681–Q682, Q688, Q69–Q74). To replicate results from previous studies (Vrijheid et al. 2011), we also considered eight subgroups of cardiac anomalies: transposition of great vessels (Q203), ventricular septal defect (Q210), atrial septal defect (Q211), atrioventricular defect (Q212), tetralogy of Fallot (Q213), tricuspid atresia and stenosis (Q224), pulmonary valve stenosis (Q221), and coarctation of aorta (Q251).

*Exposure assessment*. This study combined the spatial land use regression (LUR) modeling developed in the European Study of Cohorts for Air Pollution Effects–ESCAPE (ESCAPE 2009) with a temporal adjustment, providing both spatial and spatiotemporal exposure estimates. LUR models are commonly used to explain the small-scale within-city variation of traffic-related air pollutants; temporal adjustment is needed to account for the timing of exposure in relation to organogenesis—during weeks 3 to 8 of pregnancy (Moore et al.1998).

*Spatial estimates*. LUR models were constructed in Barcelona for the year 2009 as part of the ESCAPE project. Pollutants included NO_2_, nitrogen oxides (NO_x_), PM_10_, PM_coarse_ (particulate matter with aerodynamic diameter 10–2.5 μm), PM_2.5_, and PM_2.5_ absorbance (as a marker of black carbon) (Cyrys et al. 2003). The methodology has been described in detail elsewhere (Beelen et al. 2013; Cyrys et al. 2012; Eeftens et al. 2012a). All addresses were geocoded to assign the spatial exposure estimates. We assumed that the residential address at delivery was constant during the whole pregnancy period and that the city spatial distribution of pollutants and their determinants remained constant over the study period.

*Spatiotemporal estimates*. The start and end dates of weeks 3–8 of pregnancy were determined from the date of birth and the GA recorded on the register (*n* = 4,866). When GA was not available, the declared date of LMP was used (*n* = 261) to determine it. When only the birth date was available, we imputed the GA with the mean GA for the specific type of delivery (live birth, stillbirth, or termination of pregnancy; *n* = 111).

To estimate exposure during the “critical window” for organogenesis (weeks 3–8), background routine daily ambient air pollution monitoring data were used. NO_2_ and NO_x_ series were available for the complete study period, from 1994 through 2006 (29% of days with no measurement), and PM_10_ data from 2000 through 2006 (68% of days with no measurement). On days with no measurement, NO_2_ and NO_x_ data were estimated using the multiple imputation method developed in the ICE function of the statistical package STATA version 8 (StataCorp, College Station, TX, USA). Because of the large number of days for which PM_10_ data were missing, we did not impute missing PM_10_ data. Instead, we restricted our population to those pregnancies with data available on ≥ 2 days per week for at least 5 of the 6 weeks of interest (weeks 3–8 of gestation). This resulted in 63% of the original population. Then, according to the ratio method procedure in the ESCAPE manual for extrapolation back in time (ESCAPE 2009), we calculated the spatiotemporal exposure estimates as follow:

*Procedure for extrapolation back in time*. The daily spatiotemporal exposure estimates (*C*_extrapolated_) for each pregnancy were the spatial exposure estimation (*C*_ESCAPE_) multiplied by a daily temporal adjustment (Ratio_routine_) that was calculated as the ratio between the daily NO_2_, NO_x_, or PM_10_ measurements at the background routine monitoring station (*C*_daily_) and its annual 2009 average (*C*_routine_yearESCAPE_):

*C*_extrapolated_ = *C*_ESCAPE_ × Ratio_routine_, [1]

where

Ratio_routine_ = *C*_daily_/*C*_routine_yearESCAPE_. [2]

Daily spatiotemporal estimates were averaged over weeks 3–8 of each pregnancy to obtain the final exposure. We further applied the NO_x_ daily temporal adjustment to PM_2.5_ absorbance spatial estimates because of their high correlation (Cyrys et al. 2003), and the PM_10_ daily temporal adjustment to PM_coarse_ and PM_2.5_ (Pedersen et al. 2013), to obtain the respective spatiotemporal estimates of exposure to air pollution, following the methodology in ESCAPE (2009).

*Statistical analysis*. We examined the association between the spatial and spatiotemporal exposure estimates and 18 groups of congenital anomalies using unconditional logistic regression models. Air pollution exposure estimates were entered into the model as continuous covariates. The odds ratios (ORs) were calculated for an interquartile range (IQR) increase in air pollutant concentration measured at the spatial level to ensure consistent exposure contrasts between spatial and spatiotemporal estimates, and comparable contrasts among different pollutants. Models were adjusted for year of birth/termination, season of conception, and maternal age, all as categorical variables, and for the MEDEA socioeconomic deprivation index as a continuous variable (Dadvand et al. 2011a; Ritz et al. 2002).

We tested potential confounding by maternal smoking and maternal education in sensitivity analyses restricted to the populations with data available on these variables (70% and 80% of the full study population, respectively). Information on maternal smoking during each month of pregnancy was used to calculate the proportion of smokers during the first trimester as a two-category variable (yes or quit/no). Maternal education was coded as a four-category variable (primary/secondary/college/university). The assumption of a stable spatial exposure surface over the entire study period was investigated by stratifying the population according to year of birth/termination (before and after 2000). All analyses were performed using the statistical package STATA versions 8 and 10.1 (StataCorp).

## Results

The study included 2,247 cases and 2,991 controls. There were no statistically significant differences between cases and controls for season of conception, sex, maternal age, and socioeconomic index (Table 1), but differences were seen for maternal smoking during first trimester and for maternal education: Mothers of cases had less education and smoked more frequently than did mothers of controls. Both these characteristics were derived from the interview with the mother completed by the 35% of cases and 95% of controls; in that subpopulation, the exposure distribution was not differential among cases and controls (see Supplemental Material, Table S1).

**Table 1:** Main characteristics of cases and controls for 1994–2006 [*n* (%)].

Characteristic	Controls	Cases	*p*-Value^*a*^
Subjects	2,991 (100)	2,247 (100)
Year of birth/termination	0.8
1994	228 (7.6)	189 (8.4)
1995	220 (7.4)	152 (6.8)
1996	211 (7.1)	142 (6.3)
1997	217 (7.3)	164 (7.3)
1998	224 (7.5)	167 (7.4)
1999	224 (7.5)	187 (8.3)
2000	222 (7.4)	184 (8.2)
2001	227 (7.6)	176 (7.8)
2002	246 (8.2)	182 (8.1)
2003	231 (7.7)	163 (7.3)
2004	249 (8.3)	169 (7.5)
2005	250 (8.4)	199 (8.9)
2006	242 (8.1)	173 (7.7)
Season of conception	0.1
Winter	789 (26.4)	524 (23.3)
Spring	727 (24.3)	565 (25.1)
Summer	730 (24.4)	577 (25.7)
Autumn	745 (24.9)	581 (25.9)
Sex	0.6
Male	1,540 (53.3)	1,125 (52.0)
Female	1,348 (46.7)	1,040 (48.0)
Missing	105 (3.4)	84 (3.6)
Type of birth
Live birth	2,991 (100.0)	1,687 (75.1)
Stillbirth		179 (8.0)
Termination of pregnancy		381 (17.0)
Maternal age (years)	0.6
< 25	298 (10.4)	207 (9.5)
25–30	797 (27.8)	589 (27.1)
30–35	1,099 (38.3)	866 (39.9)
> 35	675 (23.5)	511 (23.5)
Missing	122 (4.1)	74 (3.3)
Maternal education	0.00
Primary school	103 (3.6)	130 (9.5)
Secondary school	699 (24.6)	388 (28.3)
College	816 (28.7)	289 (21.1)	
University	1,222 (43.0)	564 (41.1)
Missing	151 (5.3)	876 (63.9)
Smoking 1st trimester	0.00
No	1,767 (62.4)	481 (52.3)
Yes or quit	1,066 (35.6)	438 (47.7)
Missing	158 (5.3)	1,328 (59.1)
MEDEA index^*b*^
Mean ± SD	0.02 ± 1.05	–0.01 ± 1.00	0.2
^***a***^Chi-square test for equal distribution of categorical variables; *t*-test for equality of means of continuous variables, ­significant at α = 0.05. ^***b***^Socioeconomic index at census-tract level; the lower the MEDEA index, the more deprived the census tract.			

Exposure distributions of the air pollutants are presented in Table 2. The IQRs of the spatiotemporal exposure estimates, which were averaged over the 6-week period of organogenesis for each observation, were wider than the IQRs of the spatial estimates, which were averaged over an entire year, because of the short averaging time and seasonal variation. Exposure estimates were somewhat higher among controls than among cases for all exposures. The range of correlations between spatial exposures (*r* = 0.16–0.93) was much wider than the corresponding range of correlations among the spatiotemporal exposures (*r* = 0.85–0.91) (Table 3) because of the high seasonal correlation between the pollutants (data not shown) and the use of NO_x_ and PM_10_ temporal trends to adjust the other air pollutants. Correlations between spatial and spatiotemporal estimates were moderate to low (*r* = 0.55 and 0.26 for NO_x_ and PM_10_ respectively).

**Table 2:** Median (IQR) of spatial and spatiotemporal exposure of air pollutants, by cases and controls.

Pollutant	All median (IQR)	Controls median (IQR)	Cases median (IQR)
Spatial exposure^*a*^
NO_2_ (μ/m^3^)	55.7 (12.2)	56.0 (11.8)*	55.4 (12.7)*
NO_x_ (μ/m^3^)	88.7 (27.1)	89.8 (27.5)*	87.1 (27.2)*
PM_10_ (μ/m^3^)	38.7 (3.0)	38.8 (3.0)	38.7 (2.8)
PM_coarse_ (μ/m^3^)	21.7 (3.6)	21.7 (3.6)	21.7 (3.8)
PM_2.5_ (μ/m^3^)	16.6 (2.6)	16.6 (2.5)	16.5 (2.7)
PM_2.5_ absorbance (10^–5^/m)	2.65 (0.73)	2.68 (0.71)*	2.62 (0.75)*
Spatiotemporal exposure
1994–2006^*a*^			
NO_2_ (μ/m^3^)	53.6 (26.4)	54.1 (26.2)**	53.0 (26.3)**
NO_x_ (μ/m^3^)	110.5 (78.1)	111.5 (77.4)**	108.5 (78.7)**
PM_2.5_ absorbance (10^–5^/m)	3.21 (2.15)	3.24 (2.14)**	3.18 (2.19)**
2000–2006^*b*^			
PM_10_ (μ/m^3^)	38.6 (12.1)	38.3 (11.9)	38.9 (12.4)
PM_coarse_ (μ/m^3^)	21.1 (7.0)	21.1 (6.7)	21.1 (7.4)
PM_2.5_ (μ/m^3^)	16.6 (6.0)	16.6 (5.9)	16.6 (6.0)
^***a***^All subjects *n *= 5,238; controls *n *= 2,991; cases *n *= 2,247. ^***b***^All subjects *n *= 1,665; controls *n *= 941; cases *n *= 724. ­*Rank-sum test for difference in the distribution between cases and controls, significant at α = 0.05. **Rank-sum test for ­difference in the distribution between two populations, significant at α = 0.10.			

**Table 3:** Correlation coefficients^*a*^ between spatial and spatiotemporal exposure estimates of air pollutants (1994–2006 for NO_2_, NO_x_, and PM_2.5_ absorbance; 2000–2006 for PM_10_, PM_coarse_, and PM_2.5_).

Pollutant	NO_2_	NO_x_	PM_10_	PM_coarse_	PM_2.5_	PM_2.5_ absorbance
Spatial exposure (*n* = 5,238)
NO_x_	0.92
PM_10_	0.66	0.69
PM_coarse_	0.17	0.16	0.34
PM_2.5_	0.67	0.67	0.64	0.32
PM_2.5_ absorbance	0.93	0.92	0.68	0.17	0.70
Spatiotemporal exposure, 1994–2006 (*n *= 5,238)
NO_2_	0.53^*a*^
NO_x_	0.86	0.55^*a*^
PM_2.5_ absorbance	0.85	0.97				0.46^*a*^
Spatiotemporal exposure, 2000–2006 (*n *= 1,665)
PM_10_			0.26^*b*^
PM_coarse_			0.89	0.41^*b*^
PM_2.5_			0.91	0.86	0.47^*b*^
^***a***^Correlation between spatial and spatiotemporal exposure for each pollutant. ^***b***^Spearman, significant at α = 0.05.						

We observed several statistically significant associations between IQR increases in spatial air pollutant exposure estimates and congenital anomaly groups (see Supplemental Material, Table S2): coarctation of the aorta and NO_2_ [adjusted (adj)OR_spatial_ = 1.08; 95% CI: 1.00, 1.16], NO_x_ (adjOR_spatial_ = 1.06; 95%CI: 1.00, 1.13), and PM_2.5_ (adjOR_spatial_ = 1.25; 95% CI: 1.00, 1.57); transposition of great vessels and PM_10_ (adjOR_spatial_ = 1.27; 95% CI: 1.03, 1.57); digestive system defects and NO_2_ (adjOR_spatial_ = 1.07; 95% CI: 1.02, 1.13) and NO_x_ (adjOR_spatial_ = 1.06; 95% CI: 1.01, 1.10); and abdominal wall defects and PM_coarse_ (adjOR_spatial_ = 1.60; 95% CI: 1.04, 2.48). Furthermore, statistically significant negative associations with IQR increases in exposure were observed for all nonchromosomal anomalies and PM_2.5_ absorbance (adjOR_spatial_ = 0.92; 95% CI: 0.86, 0.98), all congenital heart defects and PM_2.5_ absorbance (adjOR_spatial_ = 0.91; 95% CI: 0.83, 0.99), and atrial septal defects and PM_coarse_ (adjOR_spatial_ = 0.73; 95% CI: 0.60, 0.89).

The association between an IQR increase in NO_2_ (12.2 μg/m^3^) and coarctation of the aorta was increased in the spatiotemporal exposure model (adjOR_spatiotemporal_ = 1.15; 95% CI: 1.01, 1.31) compared with the spatial model, though it was somewhat reduced with an IQR increase in NO_x_ (27.1 μg/m^3^; adjOR_spatiotemporal_ = 1.02; 95% CI: 1.00, 1.04) (Table 4). Similarly, digestive system defects showed a somewhat increased association with NO_2_ (adjOR_spatiotemporal_ = 1.11; 95% CI: 1.00, 1.23) and decreased association with NO_x_ (adjOR_spatiotemporal_ = 1.02; 95% CI: 1.00, 1.04). The association between abdominal wall defects and an IQR increase in PM_coarse_ (3.6 μg/m^3^) was also higher based on the spatiotemporal model (adjOR_spatiotemporal_ = 1.93; 95% CI: 1.37, 2.73) (Table 5) compared with the spatial model. The spatiotemporal model also generated several statistically significant associations that were not observed in the spatial model, including significant positive associations between abdominal wall defects and PM_10_ and PM_2.5_, and significant negative associations for ventricular septal defects and PM_10_, PM_coarse_, and PM_2.5_.

**Table 4:** Spatiotemporal exposure to NO_2_, NO_x_, and PM_2.5_ absorbance (1994–2006): adjusted OR^*a*^ (95% CI) for each IQR increase in exposure.^*b*^

Congenital anomaly	*n* Cases/*n* controls^*c*^	NO_2_	NO_x_	PM_2.5_ absorbance
All cases	2,173/2,869	0.98 (0.92,1.04)	1.00 (0.99, 1.01)	0.95 (0.86, 1.04)
Neural tube defects	139/2,869	1.01(0.83,1.22)	1.00 (0.96, 1.03)	1.09 (0.81, 1.47)
Congenital heart disease	823/2,869	0.97 (0.89, 1.06)	1.00 (0.98, 1.01)	0.94 (0.83, 1.08)
Transposition of great vessels (complete)	69/2,869	1.09 (0.93, 1.27)	1.01 (0.98, 1.04)	1.29 (0.88, 1.89)
Ventricular septal defect	351/2,869	0.98 (0.86, 1.11)	0.99 (0.97, 1.02)	0.98 (0.81, 1.19)
Atrial septal defect	229/2,869	0.94 (0.79,1.11)	0.98 (0.95, 1.02)	0.95 (0.76, 1.20)
Atrioventricular defect	30/2,650	1.03 (0.77, 1.38)	1.00 (0.96, 1.06)	1.29 (0.70, 2.37)
Tetralogy of Fallot	49/2,650	0.96 (0.68, 1.35)	0.99 (0.93, 1.06)	0.92 (0.55, 1.52)
Tricuspid atresia and stenosis	59/2,168	0.94 (0.67, 1.33)	0.98 (0.91, 1.05)	0.97 (0.61, 1.53)
Pulmonary valve stenosis	70/2,869	0.95 (0.72, 1.27)	1.00 (0.95, 1.05)	1.08 (0.72, 1.63)
Coarctation of aorta	69/2,869	1.15* (1.01, 1.31)	1.02* (1.00, 1.04)	1.28 (0.86, 1.90)
Respiratory system	138/2,869	1.01 (0.85, 1.20)	1.00 (0.97, 1.03)	1.06 (0.79, 1.44)
Orofacial clefts	130/2,869	1.08 (0.93, 1.25)	1.00 (0.97, 1.03)	1.19 (0.89, 1.59)
Digestive system	191/2,869	1.11* (1.00, 1.23)	1.02* (1.00, 1.04)	0.95 (0.74, 1.22)
Abdominal wall defects	55/2,869	0.86 (0.58, 1.28)	0.97 (0.89, 1.05)	0.71 (0.42, 1.19)
Urinary system	494/2,869	0.95 (0.85, 1.07)	0.99 (0.97, 1.01)	0.99 (0.84, 1.17)
Hypospadias	74/2,423	1.02 (0.78, 1.34)	0.98 (0.91, 1.05)	0.89 (0.57, 1.39)
Limb reduction	308/2,869	0.97 (0.85, 1.12)	0.99 (0.97, 1.02)	0.88 (0.71, 1.09)
^***a***^Model adjusted for maternal age, conception season, year of birth/termination, socioeconomic index. ^***b***^Spatial exposure IQRs for all subjects combined: NO_2_ (12.2), NO_x_ (27.1), PM_10_ (3.0), PM_coarse_ (3.6), PM_2.5_ (2.6), PM_2.5_ absorbance (0.73). ^***c***^The number of controls varies to avoid collinearity when there were no cases in certain years. *OR significant at α = 0.05.				

**Table 5:** Spatiotemporal exposure to PM_10_, PM_coarse_, and PM_2.5_ (2000–2006): adjusted OR^*a*^ (95% CI) for each IQR increase in exposure.^*b*^

Congenital anomaly	*n* Cases/*n* controls^*c*^	PM_10_	PM_coarse_	PM_2.5_
All cases	695/903	1.00 (0.96, 1.04)	1.01 (0.93, 1.10)	0.97 (0.91, 1.04)
Neural tube defects	51/903	1.01 (0.89, 1.15)	1.08 (0.83, 1.39)	1.06 (0.86, 1.30)
Congenital heart disease	248/903	0.97 (0.91, 1.03)	0.94 (0.83, 1.07)	0.93 (0.84, 1.04)
Transposition of great vessels (complete)	26/890	1.03 (0.86, 1.24)	1.07 (0.75, 1.54)	0.99 (0.73, 1.33)
Ventricular septal defect	106/903	0.88* (0.81, 0.97)	0.79* (0.66, 0.94)	0.83* (0.72, 0.97)
Atrial septal defect	58/903	0.65 (0.84, 1.09)	0.84 (0.65, 1.09)	0.88 (0.72, 1.09)
Atrioventricular defect	13/903	0.93 (0.72, 1.20)	0.64 (0.36, 1.14)	0.83 (0.54, 1.28)
Tetragy of Fallot	17/890	0.90 (0.73, 1.12)	0.86 (0.57, 1.30)	1.07 (0.76, 1.49)
Tricuspid atresia and stenosis	10/533	1.04 (0.76, 1.42)	1.26 (0.67, 2.34)	1.20 (0.74, 1.96)
Pulmonary valve stenosis	19/980	0.95 (0.76, 1.18)	1.03 (0.65, 1.61)	0.97 (0.68, 1.39)
Coarctation of aorta	28/890	0.99 (0.83, 1.18)	1.25 (0.88, 1.79)	1.08 (0.82, 1.42)
Respiratory	49/903	1.10 (0.98, 1.25)	1.14 (0.89, 1.47)	1.12 (0.92, 1.36)
Orofacial clefts	40/903	0.98 (0.85, 1.13)	0.92 (0.70, 1.22)	0.99 (0.79, 1.24)
Digestive system	47/890	0.99 (0.87, 1.12)	1.09 (0.84, 1.42)	1.03 (0.83, 1.27)
Abdominal wall defects	22/890	1.36* (1.14, 1.62)	1.93* (1.37, 2.73)	1.33* (1.00, 1.76)
Urinary	179/903	1.00 (0.93, 1.07)	0.96 (0.83, 1.10)	0.96 (0.85, 1.07)
Hypospadias	23/700	1.15 (0.94, 1.41)	1.15 (0.77, 1.70)	1.18 (0.87, 1.60)
Limb reduction	109/903	0.94 (0.86, 1.03)	0.90 (0.75, 1.08)	0.92 (0.79, 1.06)
^***a***^Model adjusted for maternal age, conception season, year of birth/termination, socioeconomic index. ^***b***^Spatial exposure IQRs for all subjects combined: NO_2_ (12.2), NO_x_ (27.1), PM_10_ (3.0), PM_coarse_ (3.6), PM_2.5_ (2.6), PM_2.5_ absorbance (0.73).^***c***^The number of controls varies to avoid collinearity when there were no cases in certain years. *OR significant at α = 0.05.				

Associations based on spatial models adjusted for maternal smoking (*n* = 3,752) or maternal education (*n* = 4,211) were consistent with estimates that were not adjusted for these variables when restricted to the population with available smoking or education data (see Supplemental Material, Tables S3 and S4). However, some associations differed when based on the restricted populations compared with the full study population. In particular, there were more positive associations (ORs > 1) in the restricted population. The direction and magnitude of associations were comparable for anomalies identified from 1994 through 1999 (*n* = 2,324) and 2000 through 2006 (*n* = 2,913), consistent with our assumption of a stable exposure surface over the entire study period, though estimates based on the earlier subset were less precise (see Supplemental Material, Table S5).

## Discussion

In this study we used both spatial and spatiotemporal exposure assessment frameworks to evaluate the association between traffic-related air pollution and groups of congenital anomalies. We included pollutants not previously studied in this field: PM_coarse_, PM_2.5_, and PM_2.5_ absorbance, all of which are characterized by within-city variability (Eeftens et al. 2012b; Jerrett et al. 2005). We found little evidence for an association between most of the traffic-related air pollutants and most of the congenital anomaly groups studied. However, we observed statistically significant positive associations with both spatial and spatiotemporal exposure estimates for NO_2_ and NO_x_ with coarctation of the aorta and digestive system anomalies, and for PM_coarse_ and abdominal wall defects.

Given the multiple comparisons involved in testing a large range of congenital anomaly subtypes against six pollution measures, our analyses are likely to have given rise to some chance associations, and results should be interpreted with caution. To avoid errors due to false-positive statistically significant associations (type 1 errors), we emphasized statistically significant associations from the spatial model that persisted after temporal adjustment, in contrast with statistically significant positive or negative associations based on only one of the two exposure models, which we considered more likely to be attributable to chance. Evaluation of consistency with the previous meta-analysis has further been used to interpret the role of chance as an explanation of our main findings.

The positive association between coarctation of the aorta and NO_2_ is consistent with a recent meta-analysis (Vrijheid et al. 2011) that combined results of four published studies and reported a summary OR for coarctation of the aorta of 1.20 (95% CI: 1.00, 1.44) per 10-ppb increase in NO_2_ exposure. For comparison, in our study the OR from the spatiotemporal model for a 10-ppb increment in NO_2_ was 1.23 (95% CI: 1.02, 1.48). Although in our study the number of cases with coarctation of the aorta is small (*n* = 69), the consistency of findings between spatial and spatiotemporal models, and with the published meta-analysis, appears to strengthen evidence for an association. NO_2_ and NO_x_ were highly correlated, making it hard to separate their effect, but in the spatiotemporal exposure models NO_2_ appeared somewhat more strongly associated with coarctation of the aorta than NO_x_. We also found consistent results between the spatial and the spatiotemporal models when analyzing the association between NO_2_, NO_x_, and digestive system anomalies. Only two previous studies evaluated these anomalies: Dolk et al. (2010) reported no association with NO_2_, but the exposure assessment was based on annual mean levels only; Rankin et al. (2009) evaluated digestive system anomalies in relation to black smoke and also reported no association. In the present study, an association between abdominal wall defects and PM_coarse_ was observed in both spatial and spatiotemporal models. We are aware of only two previous studies that evaluated associations between air pollutants and abdominal wall defects (Dolk et al. 2010; Padula et al. 2013a), and both reported nonsignificant associations except for the association between PM_10_ and omphalocele reported by Dolk et al. (2010). Therefore, further research on this outcome is needed.

The exposure assessment method used in this study was a temporally adjusted LUR built to capture and replicate the spatial variability within cities of traffic-related air pollutants (Cyrys et al. 2012; Eeftens et al. 2012b). Additionally, for congenital anomalies, it is of great importance to assess exposure during the critical pregnancy weeks 3–8 (Moore et al. 1998), because any exposure after this period would not contribute to the etiology of a major congenital anomaly. Only recent studies on congenital anomalies and air pollution used a spatiotemporal exposure assessment, but they were not covering the same range of pollutants (Dadvand et al. 2011b) or assessing the exposure at less refined spatial resolution (Agay-Shay et al. 2013; Padula et al. 2013a, 2013b). The spatial LUR model we used for Barcelona resulted in a spatial exposure assessment with similar variability (i.e., similar width of the IQR) to previous studies in this field (Vrijheid et al. 2011). After the temporal adjustment, the exposure variation increased, widening the IQR by > 50% for all pollutants (Table 2), thus giving greater statistical power to detect associations in the spatiotemporal models compared with the spatial models. In particular, NO_2_ and NO_x_ IQRs (reflecting within-city spatial variation) were larger than for particulate matter, partially explaining the null results for particulate matter in the spatial exposure models. One important assumption in using the LUR is that the city spatial distribution of pollutants and their determinants remained constant over the study period; if this assumption is violated, the ORs may be attenuated (Wu et al. 2011). To test this assumption we split the population by earlier and later years of termination/birth (before 2000 vs. during or after 2000); results were consistent with the overall analyses, although earlier years had wider confidence intervals than the later years. Recently Cesaroni et al. (2012) showed in Rome, a city with characteristics (e.g., climate, traffic) similar to those of Barcelona, a correlation of 96% for the exposure estimates from two LUR models produced 11 years apart. Furthermore, we did not observe long-term trends in NO_2_ and PM_10_ (data not shown). A limitation of our exposure assessment method is that we used NO_x_ and PM_10_ temporal trends to adjust other pollutant exposure estimates. This led to high correlations between the spatiotemporal estimates of these pollutants and thus difficulty in the interpretation of results from these pollutants independently. However, we followed the approach developed in the ESCAPE protocol, which has already been used in the recent publication on air pollution effect on pregnancy outcomes by Pedersen et al. (2013). Another exposure limitation was related to the missing data on the PM_10_ temporal series, which led to a loss of statistical power in the spatiotemporal models of pollutants temporally adjusted with that series (PM_10_, PM_coarse_, and PM_2.5_).

Exposure misclassification could be attributable to the time spent in different environments, although we think that it is unlikely to be differential among cases and controls. Exposure misclassification could further have arisen because we estimated outdoor exposure at the residential address at termination/birth as a surrogate of the personal exposure during the first months of pregnancy. In our recent study in Barcelona (Schembari et al. 2013) among 54 pregnant women who carried a personal PM_2.5_ sampler for 2 days and NO_x_/NO_2_ passive badges for 1 week, pregnant women reported time spent at home to be around 60–70% per day, and the correlation between personal exposure and outdoors levels of air pollution ranged from 0.39 for PM_2.5_ and 0.78 for NO_x_. This suggests that, particularly for the latter, ambient outdoor levels could act as a good surrogate for personal exposure levels. Residual misclassification may have occurred if women changed residences during pregnancy; but this is unlikely to occur differentially among cases and controls, accordingly with results published by Miller et al. (2010). Residential mobility was estimated to be between 1% and 6% in a recent Spanish study based on four birth cohorts (Estarlich et al. 2011).

We examined a wide range of congenital anomalies that were subtyped following the classification proposed by EUROCAT (2009). The REDCB includes cases identified among live births, stillbirths, and termination of pregnancy following prenatal diagnosis, which is particularly important (Ritz 2010) for some severe congenital anomalies such as neural tube defects or congenital heart disease (27% and 22.6% of anomalies, respectively, in our study). The register follows the EUROCAT guidelines, but ascertainment may be incomplete if the congenital anomaly is not diagnosed prenatally or at birth. Therefore, the prevalence of all congenital anomalies (including live births, fetal deaths, stillbirths, and termination of pregnancy) for Barcelona was lower, during 1994–2006, than the European register—184/10,000 and 233/10,000 respectively (EUROCAT 2012). The register uses active case ascertainment and simultaneous ascertainment of population-based controls; it recodes the address at delivery allowing the geocoding used for the exposure assessment in this study. We adjusted for maternal age, conception season, year of birth, and socioeconomic status, although the latter was classified at census tract level.

Only a subset of participants had data on smoking (70%) and education (80%). The sensitivity analyses on these subpopulations showed little impact of maternal smoking or education on the findings, thus indicating that their confounding effect is likely to be small even in the full study population. Nevertheless, relying on these subpopulation could be misleading: Missing information probably did not occur at random among cases and controls mainly because mothers of cases detected after birth were not interviewed. This may constitute a selective population due to the fact that only specific congenital anomalies are detected after birth; for example, congenital heart defects are frequently detected in the first year of life (Todros et al. 2001). The results of the sensitivity analyses differed for some congenital anomalies/exposure from those on the full population; in particular, PM_10_ was statistically significantly associated to congenital heart defects in the smoking subpopulation (both adjusted and unadjusted), but not in the main analyses nor in the one adjusted for maternal education. Residual confounding by unmeasured risk factors such as alcohol consumption and dietary factors (e.g., folic acid) could have influenced our findings. However, it is unlikely that such factors would be strongly related to air pollution exposure, and we can partially acount for them by adjusting for social class on area level in the main analyses, and on individual level as sensitivity analysis.

The possible mechanisms and causes underlying the development of congenital anomalies are still unclear, because of their probable multifactorial aetiology (Ritz 2010) and because different types of anomalies are likely to have very distinct etiology. Air pollution–induced oxidative stress during pregnancy has been suggested as one possible mechanism behind pregnancy outcomes (Kannan et al. 2006; Slama et al. 2008), because it regulates the pulmonary and placental inflammation, the hemodynamic responses, and thus the transplacental oxygenation and transportation of nutrients. Oxidative stress may also affect organogenesis and neural crest cell migration and differentiation (Hassler and Moran 1986; Ritz et al. 2002), which play an important role in heart development (Keyte and Redmond Hutson 2012). More recently, van Beynum et al. (2008) suggested a possible gene–environment interaction effect leading to an increased risk of congenital heart defects in mothers exposed to nitric oxide and smoking.

## Conclusion

Our results overall do not indicate an association between exposure to traffic-related air pollution and many groups of congenital anomalies in Barcelona, even though the air pollution levels are some of the highest in Europe. The positive association of NO_2_ and NO_x_ with coarctation of the aorta is consistent with a findings of a meta-analysis of previous studies, and requires further study. Associations of digestive system anomalies with NO_2_ and NO_x_, and of abdominal wall defects with PM_coarse_, also call for confirmation.

## Supplemental Material

(229 KB) PDF
