# Supplementary material for: Traffic-Related Air Pollution and Congenital Anomalies in Barcelona
Source: Environ Health Perspect. 2014 Jan 3;122(3):317–23. doi: 10.1289/ehp.1306802 (PMC3948033; doi:10.1289/ehp.1306802)
Supplement: (229 KB) PDF [file ehp.1306802.s001.pdf]

Supplemental Material
Traffic-Related Air Pollution and Congenital Anomalies in Barcelona

Anna Schembari, Mark J. Nieuwenhuijsen, Joaquin Salvador, Audrey de Nazelle, Marta Cirach, Payam Dadvand, Rob Beelen, Gerard Hoek, Xavier Basagaña, and Martine Vrijheid

Table of contents

Supplemental Material, Table S1. Exposure median (inter-quartile range (IQR)) among cases and controls, by smoking status during first trimester and by maternal education p. 2

Supplemental Material, Table S2. Spatial exposure (main analyses) to NO_2, NO_x, PM_{10}, PM_{coarse}, $PM_{2.5}$, and $PM_{2.5}$ absorbance: adjusted odds ratio (OR)[a] and 95% confidence interval (CI) for each inter-quartile range increase in exposure p. 3

Supplemental Material, Table S3. Sensitivity analyses by maternal smoking status: Models for spatial exposure to air pollutants. p. 4

Supplemental Material, Table S4. Sensitivity analyses by maternal education: Models for spatial exposure to air pollutants. p. 6

Supplemental Material, Table S5. Stratified analyses by year of birth: Models for spatial exposure to air pollutants: adjusted odds ratio (OR)[a] and 95% confidence interval (CI) for each inter-quartile range increase[b] in exposure p. 8

Supplemental Material, Table S1. Air pollution exposure estimates [median (IQR)] by maternal smoking status during first trimester and by maternal education.

	NO_2 $\mu g/m^3$		NO_x $\mu g/m^3$		PM_{10} $\mu g/m^3$		PM_{coarse} $\mu g/m^3$		$PM_{2.5}$ $\mu g/m^3$		$PM_{2.5}$ absorbance	
	**Controls**	**Cases**	**Controls**	**Cases**	**Controls**	**Cases**	**Controls**	**Cases**	**Controls**	**Cases**	**Controls**	**Cases**
Maternal smoking during 1st trimester	Median (IQR)	Median (IQR)	Median (IQR)	Median (IQR)	Median (IQR)	Median (IQR)	Median (IQR)	Median (IQR)	Median (IQR)	Median (IQR)	Median (IQR)	Median (IQR)
No	56.2 (11.6)	56.5 (13.2)	90.2 (27.8)	87.9 (28.0)	38.9 (3.1)	38.8 (3.0)	21.7 (3.5)	21.9 (3.9)	16.7 (2.6)	16.6 (2.7)	2.7 (0.7)	2.7 (0.7)
Yes + Quit	55.5 (12.4)	55.6 (13.6)	88.8 (26.8)	87.0 (27.7)	38. (2.9)	38.6 (2.9)	21.6 (3.6)	21.8 (3.6)	16.4 (2.6)	16.5 (2.8)	2.7 (0.7)	2.6 (0.7)
Maternal education												
Primary school	53.1 (10.5)	54.6 (10.4)	82.8 (17.8)	83.2 (18.3)	38.3 (1.9)	38.5 (2.5)	22.6 (3.0)	22.8 (2.1)	15.9 (2.2)	16.1 (3.0)	2.4 (0.6)	2.6 (0.5)
Secondary school	53.8 (12.6)	54.2 (12.6)	84.3 (22.5)	84.3 (23.3)	38.5 (2.6)	38.6 (2.8)	21.8 (3.8)	22.0 (3.6)	16.2 (2.8)	16.2 (2.7)	2.5 (0.7)	2.6 (0.7)
College complete	56.2 (11.2)	56.1 (12.6)	90.9 (26.6)	88.9 (27.5)	38.9 (2.9)	38.6 (3.2)	21.7 (3.4)	21.8 (4.1)	16.6 (2.3)	16.4 (2.8)	2.7 (0.6)	2.7 (0.6)
University	57.4 (12.3)	56.9 (13.4)	92.0 (32.0)	91.1 (29.6)	39.0 (3.6)	38.9 (3.0)	21.4 (3.4)	21.6 (3.4)	17.0 (2.7)	17.0 (2.7)	2.8 (0.7)	2.7 (0.8)

[a]Smoking during first trimester was calculated as the percentage of mothers who declared that they smoked for the first, the second or the third month of pregnancy.

Supplemental Material, Table S2. Spatial exposure to NO_2, NO_x, PM_{10}, PM_{coarse}, $PM_{2.5}$, and $PM_{2.5}$ absorbance: adjusted odds ratio (OR)[a] and 95% confidence interval (CI) for each inter-quartile range increase in exposure[b].

Congenital anomaly	N Cases /N Controls[c]	NO_2 OR (95% CI)	NO_x OR (95% CI)	PM_{10} OR (95% CI)	PM_{coarse} OR (95% CI)	$PM_{2.5}$ OR (95% CI)	$PM_{2.5}$ absorbance OR (95% CI)
All non-chromosomal	2173/2869	0.98 (0.94, 1.02)	0.98 (0.95, 1.01)	0.96 (0.90, 1.01)	1.01 (0.93, 1.09)	0.95 (0.90, 1.00)	0.92* (0.86, 0.98)
Neural tube defects	139/2869	0.91 (0.77, 1.07)	0.92 (0.79, 1.07)	1.03 (0.86, 1.22)	0.90 (0.69, 1.16)	0.96 (0.81, 1.15)	0.92 (0.76, 1.12)
Congenital heart defect	823/2869	0.96 (0.90, 1.02)	0.96 (0.91, 1.02)	0.96 (0.88, 1.04)	0.91 (0.81, 1.02)	0.97 (0.90, 1.05)	0.91* (0.83, 0.99)
Transposition of great vessels (complete)	69/2869	1.05 (0.95, 1.15)	1.03 (0.94, 1.12)	1.27* (1.03, 1.57)	0.94 (0.66, 1.34)	1.14 (0.91, 1.45)	1.28 (0.99, 1.65)
Ventricular septal defect	351/2869	0.97 (0.89, 1.06)	0.96 (0.89, 1.05)	0.96 (0.85, 1.07)	0.90 (0.77, 1.07)	0.99 (0.89, 1.12)	0.98 (0.81, 1.19)
Atrial septal defect	229/2869	0.93 (0.82, 1.05)	0.92 (0.81, 1.04)	0.98 (0.85, 1.13)	0.73* (0.60, 0.89)	0.98 (0.86, 1.12)	0.91 (0.77, 1.07)
Atrio-ventricular defect	30/2869	1.01 (0.83, 1.23)	1.01 (0.87, 1.19)	1.33 (0.98, 1.82)	0.91 (0.52, 1.58)	1.05 (0.72, 1.51)	1.18 (0.79, 1.76)
Tetralogy of fallot	49/2650[c]	0.99 (0.81, 1.21)	0.92 (0.72, 1.18)	0.88 (0.64, 1.21)	0.93 (0.61, 1.42)	1.09 (0.82, 1.43)	0.90 (0.65, 1.25)
Tricuspid atresia and stenosis	59/2869	0.90 (0.69, 1.18)	0.89 (0.69, 1.14)	1.05 (0.81, 1.37)	1.36 (0.90, 2.04)	1.19 (0.93, 1.52)	0.95 (0.70, 1.30)
Pulmonary valve stenosis	70/2869	0.88 (0.69, 1.12)	0.89 (0.71, 1.12)	0.85 (0.65, 1.13)	0.97 (0.68, 1.40)	0.90 (0.70, 1.15)	0.87 (0.65, 1.15)
Coarctation of aorta	69/2869	1.08* (1.00, 1.16)	1.06* (1.00, 1.13)	1.17 (0.94, 1.46)	1.39 (0.96, 2.03)	1.25* (1.00, 1.57)	1.18 (0.91, 1.53)
Respiratory system	138/2869	0.99 (0.88, 1.11)	0.98 (0.88, 1.09)	1.11 (0.94, 1.31)	0.98 (0.76, 1.27)	1.01 (0.85, 1.20)	1.01 (0.83, 1.23)
Oro-facial clefts	130/2869	1.02 (0.93, 1.13)	1.00 (0.91, 1.10)	0.99 (0.83, 1.19)	0.98 (0.75, 1.28)	1.02 (0.86, 1.22)	1.11 (0.91, 1.35)
Digestive system	191/2869	1.07* (1.02, 1.13)	1.06* (1.01, 1.10)	1.06 (0.91, 1.22)	1.14 (0.91, 1.42)	1.07 (0.92, 1.23)	0.97 (0.82, 1.15)
Abdominal wall defects	55/2869	1.01 (0.85, 1.20)	1.00 (0.85, 1.17)	1.07 (0.82, 1.40)	1.60* (1.04, 2.48)	0.94 (0.71, 1.24)	0.94 (0.69, 1.29)
Urinary system	494/2869	0.99 (0.92, 1.05)	0.97 (0.91, 1.03)	0.96 (0.87, 1.06)	0.97 (0.84, 1.11)	0.98 (0.89, 1.08)	0.97 (0.87, 1.08)
Hypospadias	74/2869	0.99 (0.82, 1.20)	0.96 (0.79, 1.16)	1.01 (0.79, 1.29)	1.37 (0.96, 1.97)	1.01 (0.80, 1.28)	1.00 (0.76, 1.31)
Limb reduction	308/2869	0.98 (0.89, 1.07)	0.99 (0.92, 1.06)	0.96 (0.85, 1.09)	1.01 (0.84, 1.21)	0.96 (0.85, 1.08)	0.88 (0.77, 1.01)

[a] Models adjusted for maternal age, conception season, year of birth/termination, socio economic index. [b] Spatial Spatial exposure inter-quartile ranges for all subjects combined: NO_2 (12.2), NO_x (27.1), PM_{10} (3.0), PM_{coarse} (3.6), $PM_{2.5}$ (2.6), $PM_{2.5}$ absorbance (0.73). [c]The number of controls varies to avoid collinearity when there were no cases in certain years. *Odds ratio significant at α level ($\alpha = 0.05$).

Supplemental Material, Table S3. Sensitivity analyses by maternal smoking status: Models for spatial exposure to air pollutants. Adjusted odds ratio (OR) and 95% confidence interval (CI) for each inter-quartile range increase[a] in exposure.

Congenital anomaly	Model[b]	N cases/ N controls[c]	NO_2 OR (95% CI)	NO_x OR (95% CI)	PM_{10} OR (95% CI)	PM_{coarse} OR (95% CI)	$PM_{2.5}$ OR (95% CI)	$PM_{2.5}$ absorbance OR (95% CI)
All non-chromosomal	1	891/2717	1.01 (0.96, 1.06)	1.00 (0.96, 1.04)	1.01 (0.94, 1.10)	1.09 (0.97, 1.22)	0.99 (0.91, 1.07)	0.98 (0.90, 1.07)
All non-chromosomal	2	891/2717	1.01 (0.97, 1.06)	1.00 (0.96, 1.04)	1.03 (0.95, 1.11)	1.09 (0.98, 1.23)	1.00 (0.93, 1.08)	1.00 (0.91, 1.09)
Neural Tube Defects	1	74/2717	0.94 (0.76, 1.16)	0.96 (0.81, 1.15)	0.96 (0.75, 1.23)	0.85 (0.60, 1.20)	0.86 (0.68, 1.10)	0.96 (0.74, 1.26)
Neural Tube Defects	2	74/2717	0.94 (0.76, 1.16)	0.96 (0.81, 1.15)	0.96 (0.75, 1.23)	0.85 (0.60, 1.20)	0.86 (0.68, 1.09)	0.96 (0.73, 1.25)
Congenital heart defect	1	231/2717	1.00 (0.92, 1.09)	0.99 (0.91, 1.07)	1.16 (1.02, 1.32)	1.19 (0.96, 1.46)	1.16 (1.02, 1.33)	1.07 (0.91, 1.24)
Congenital heart defect	2	231/2717	1.01 (0.93, 1.10)	0.99 (0.92, 1.08)	1.19 (1.04, 1.35)	1.20 (0.98, 1.49)	1.19 (1.04, 1.36)	1.09 (0.94, 1.27)
Transposition of great vessels (complete)	1	38/2518	1.04 (0.91, 1.18)	1.02 (0.90, 1.15)	1.22 (0.91, 1.62)	0.93 (0.58, 1.51)	1.08 (0.78, 1.48)	1.31 (0.93, 1.85)
Transposition of great vessels (complete)	2	38/2518	1.04 (0.92, 1.18)	1.02 (0.91, 1.15)	1.23 (0.92, 1.64)	0.94 (0.58, 1.52)	1.09 (0.79, 1.50)	1.33 (0.94, 1.87)
Ventricular septal defect	1	96/2717	1.06 (0.97, 1.15)	1.02 (0.94, 1.12)	1.27 (1.06, 1.52)	1.14 (0.83, 1.56)	1.21 (0.99, 1.47)	1.33 (1.07, 1.66)
Ventricular septal defect	2	96/2717	1.06 (0.97, 1.15)	1.03 (0.94, 1.12)	1.29 (1.08, 1.54)	1.15 (0.84, 1.58)	1.23 (1.01, 1.509	1.35 (1.08, 1.69)
Atrial septal defect	1	51/2717	1.06 (0.94, 1.18)	1.04 (0.94, 1.15)	1.36 (1.08, 1.72)	0.84 (0.56, 1.26)	1.39 (1.08, 1.80)	1.21 (0.89, 1.65)
Atrial septal defect	2	51/2717	1.06 (0.94, 1.18)	1.04 (0.94, 1.15)	1.38 (1.09, 1.75)	0.85 (0.57, 1.28)	1.42 (1.10, 1.83)	1.24 (0.91, 1.68)
Atrioventricular defect	1	13/2291	0.91 (0.54, 1.55)	0.99 (0.74, 1.34)	1.34 (0.84, 2.15)	1.09 (0.46, 2.54)	0.91 (0.50, 1.63)	1.00 (0.53, 1.88)
Atrioventricular defect	2	13/2291	0.92 (0.54, 1.56)	1.00 (0.74, 1.35)	1.36 (0.84, 2.20)	1.09 (0.46, 2.55)	0.92 (0.51, 1.66)	1.01 (0.53, 1.91)
Tetragy of fallot	1	21/2281	0.99 (0.75, 1.31)	0.86 (0.57, 1.29)	0.93 (0.59, 1.49)	1.19 (0.61, 2.33)	1.04 (0.67, 1.60)	0.91 (0.56, 1.49)
Tetragy of fallot	2	21/2281	1.00 (0.76, 1.32)	0.87 (0.58, 1.31)	0.95 (0.60, 1.53)	1.22 (0.63, 2.39)	1.07 (0.69, 1.65)	0.93 (0.56, 1.53)
Tricuspid atresia and stenosis	1	15/1454	1.07 (0.90, 1.28)	1.07 (0.95, 1.21)	1.49 (0.99, 2.24)	2.84 (1.14, 7.08)	1.26 (0.77, 2.07)	1.66 (0.97, 2.84)

Congenital anomaly	Model[b]	N cases/ N controls[c]	NO_2 OR (95% CI)	NO_x OR (95% CI)	PM_{10} OR (95% CI)	PM_{coarse} OR (95% CI)	$PM_{2.5}$ OR (95% CI)	$PM_{2.5}$ absorbance OR (95% CI)
Tricuspid atresia and stenosis	2	15/1454	1.07 (0.90, 1.28)	1.07 (0.95, 1.21)	1.52 (1.01, 2.29)	2.93 (1.16, 7.40)	1.31 (0.80, 2.16)	1.70 (0.98, 2.94)
Pulmonary valve stenosis	1	13/1878	0.98 (0.71, 1.36)	0.85 (0.50, 1.44)	1.04 (0.60, 1.79)	1.05 (0.45, 2.43)	1.13 (0.66, 1.93)	1.16 (0.63, 2.13)
Pulmonary valve stenosis	2	13/1878	0.98 (0.71, 1.36)	0.85 (0.51, 1.44)	1.04 (0.60, 1.79)	1.06 (0.45, 2.48)	1.14 (0.67, 1.95)	1.17 (0.64, 2.15)
Coarctation of aorta	1	13/1923	1.01 (0.78, 1.32)	1.04 (0.88, 1.22)	1.30 (0.79, 2.14)	1.49 (0.62, 3.62)	1.75 (1.07, 2.86)	1.07 (0.56, 2.02)
Coarctation of aorta	2	13/1923	1.02 (0.78, 1.32)	1.04 (0.88, 1.23)	1.32 (0.80, 2.18)	1.51 (0.62, 3.66)	1.78 (1.09, 2.91)	1.08 (0.57, 2.06)
Respiratory	1	52/2517	0.98 (0.80, 1.18)	0.98 (0.82, 1.16)	1.10 (0.85, 1.44)	0.88 (0.58, 1.33)	1.06 (0.80, 1.40)	1.00 (0.72, 1.37)
Respiratory	2	52/2517	0.99 (0.82, 1.19)	0.98 (0.83, 1.16)	1.13 (0.87, 1.47)	0.89 (0.59, 1.35)	1.09 (0.82, 1.45)	1.02 (0.74, 1.40)
Oro-facial clefts	1	91/2517	1.01 (0.89, 1.15)	0.99 (0.88, 1.12)	0.93 (0.74, 1.17)	0.94 (0.69, 1.28)	0.99 (0.80, 1.22)	1.05 (0.82, 1.33)
Oro-facial clefts	2	91/2517	1.01 (0.89, 1.15)	1.00 (0.89, 1.12)	0.94 (0.75, 1.17)	0.94 (0.69, 1.29)	1.00 (0.81, 1.23)	1.05 (0.83, 1.34)
Digestive system	1	93/2717	1.08 (1.00, 1.16)	1.06 (1.00, 1.13)	1.25 (1.04, 1.51)	1.32 (0.95, 1.82)	1.16 (0.95, 1.43)	1.12 (0.89, 1.41)
Digestive system	2	93/2717	1.08 (1.00, 1.16)	1.06 (1.00, 1.13)	1.26 (1.04, 1.51)	1.32 (0.95, 1.82)	1.17 (0.95, 1.43)	1.12 (0.89, 1.41)
Abdominal wall defects	1	26/2287	1.00 (0.77, 1.30)	1.01 (0.83, 1.23)	0.98 (0.65, 1.48)	1.75 (0.93, 3.30)	0.93 (0.63, 1.39)	0.90 (0.57, 1.43)
Abdominal wall defects	2	26/2287	1.01 (0.79, 1.29)	1.01 (0.84, 1.23)	1.01 (0.66, 1.53)	1.79 (0.95, 3.39)	0.97 (0.65, 1.45)	0.93 (0.58, 1.48)
Urinary	1	221/2717	0.99 (0.90, 1.09)	0.96 (0.87, 1.07)	1.04 (0.91, 1.20)	0.97 (0.79, 1.19)	1.00 (0.87, 1.15)	1.01 (0.86, 1.18)
Urinary	2	221/2717	1.00 (0.91, 1.10)	0.97 (0.88, 1.07)	1.06 (0.92, 1.22)	0.98 (0.79, 1.20)	1.02 (0.89, 1.18)	1.03 (0.88, 1.20)
Hypospadias	1	39/2290	0.95 (0.70, 1.28)	0.90 (0.66, 1.22)	1.05 (0.75, 1.46)	1.50 (0.91, 2.48)	0.95 (0.69, 1.31)	0.98 (0.67, 1.43)
Hypospadias	2	39/2290	0.95 (0.70, 1.28)	0.89 (0.66, 1.22)	1.04 (0.75, 1.45)	1.50 (0.91, 2.47)	0.94 (0.68, 1.30)	0.98 (0.67, 1.43)
Limb	1	151/2717	1.05 (0.98, 1.13)	1.04 (0.98, 1.10)	1.08 (0.92, 1.28)	1.18 (0.92, 1.52)	1.13 (0.96, 1.33)	1.05 (0.87, 1.27)
Limb	2	151/2717	1.06 (0.99, 1.13)	1.04 (0.98, 1.10)	1.10 (0.94, 1.30)	1.19 (0.92, 1.54)	1.16 (0.99, 1.37)	1.07 (0.89, 1.30)

[a]Spatial exposure inter-quartile ranges for all subjects combined: NO_2 (12.2), NO_x (27.1), PM_{10} (3.0), PM_{coarse} (3.6), $PM_{2.5}$ (2.6), $PM_{2.5}$ absorbance (0.73). [b]Model 1:adjusted for maternal age, conception season, year of birth, socio economic index. Model 2: Model 1 + smoking. [c]The number of controls varies to avoid collinearity when there were no cases in certain years.

Supplemental Material, Table S4. Sensitivity analyses by maternal education: Models for spatial exposure to air pollutants. Adjusted odds ratio (OR) and 95% confidence interval (CI) for each inter-quartile range increase[a] in exposure.

Congenital anomaly	Model[b]	N cases/ N controls[c]	NO_2 OR (95% CI)	NO_x OR (95% CI)	PM_{10} OR (95% CI)	PM_{coarse} OR (95% CI)	$PM_{2.5}$ OR (95% CI)	$PM_{2.5}$ absorbance OR (95% CI)
All non-chromosomal	1	1332/2725	1.00 (0.96, 1.04)	1.00 (0.96, 1.03)	0.99 (0.93, 1.07)	1.08 (0.98, 1.20)	0.98 (0.92, 1.05)	0.97 (0.90, 1.04)
All non-chromosomal	2	1332/2725	1.01 (0.97, 1.05)	1.01 (0.97, 1.04)	1.01 (0.94, 1.08)	1.06 (0.95, 1.17)	1.01 (0.94, 1.08)	1.00 (0.92, 1.08)
Neural Tube Defects	1	96/2725	0.88 (0.72, 1.08)	0.92 (0.77, 1.11)	1.05 (0.85, 1.29)	0.87 (0.64, 1.18)	0.89 (0.72, 1.11)	0.89 (0.70, 1.13)
Neural Tube Defects	2	96/2725	0.87 (0.70, 1.07)	0.92 (0.76, 1.10)	1.04 (0.85, 1.29)	0.87 (0.64, 1.18)	0.88 (0.70, 1.09)	0.87 (0.68, 1.11)
Congenital heart disease	1	397/2725	0.97 (0.88, 1.05)	0.97 (0.90, 1.05)	1.03 (0.92, 1.15)	1.00 (0.85, 1.17)	1.03 (0.92, 1.15)	0.96 (0.85, 1.09)
Congenital heart disease	2	397/2725	0.98 (0.90, 1.06)	0.98 (0.92, 1.05)	1.04 (0.93, 1.16)	0.98 (0.83, 1.15)	1.05 (0.94, 1.17)	0.99 (0.87, 1.12)
Transposition of great vessels (complete)	1	46/2725	1.03 (0.91, 1.18)	1.02 (0.90, 1.15)	1.13 (0.85, 1.49)	0.89 (0.58, 1.37)	1.12 (0.84, 1.51)	1.24 (0.90, 1.71)
Transposition of great vessels (complete)	2	46/2725	1.04 (0.92, 1.18)	1.03 (0.91, 1.15)	1.15 (0.86, 1.53)	0.86 (0.56, 1.33)	1.16 (0.86, 1.57)	1.29 (0.93, 1.80)
Ventricular septal defect	1	173/2725	1.02 (0.93, 1.12)	0.99 (0.90, 1.09)	1.06 (0.91, 1.24)	1.01 (0.80, 1.27)	1.08 (0.93, 1.27)	1.08 (0.90, 1.29)
Ventricular septal defect	2	173/2725	1.03 (0.94, 1.12)	1.01 (0.92, 1.10)	1.08 (0.92, 1.27)	0.99 (0.78, 1.25)	1.11 (0.95, 1.30)	1.12 (0.94, 1.35)
Atrial septal defect	1	89/2725	0.97 (0.82, 1.15)	0.98 (0.85, 1.13)	1.11 (0.90, 1.37)	0.73 (0.54, 0.99)	1.08 (0.88, 1.34)	0.96 (0.75, 1.23)
Atrial septal defect	2	89/2725	0.96 (0.80, 1.14)	0.97 (0.83, 1.12)	1.09 (0.89, 1.34)	0.73 (0.53, 0.99)	1.06 (0.86, 1.32)	0.94 (0.73, 1.20)
Atrioventricular defect	1	18/2725	1.00 (0.74, 1.34)	1.02 (0.83, 1.24)	1.35 (0.91, 2.00)	0.85 (0.43, 1.70)	0.92 (0.57, 1.50)	1.09 (0.64, 1.85)
Atrioventricular defect	2	18/2725	1.04 (0.81, 1.33)	1.05 (0.88, 1.24)	1.46 (0.98, 2.19)	0.86 (0.44, 1.69)	1.00 (0.63, 1.60)	1.22 (0.71, 2.10)
Tetragy of fallot	1	29/2289	1.01 (0.82, 1.24)	0.98 (0.78, 1.24)	0.88 (0.58, 1.34)	0.98 (0.57, 1.71)	1.10 (0.77, 1.59)	0.97 (0.64, 1.48)

Congenital anomaly	Model[b]	N cases/ N controls[c]	NO_2 OR (95% CI)	NO_x OR (95% CI)	PM_{10} OR (95% CI)	PM_{coarse} OR (95% CI)	$PM_{2.5}$ OR (95% CI)	$PM_{2.5}$ absorbance OR (95% CI)
Tetragy of fallot	2	29/2289	1.04 (0.88, 1.24)	1.02 (0.85, 1.22)	0.95 (0.62, 1.45)	0.93 (0.54, 1.62)	1.21 (0.84, 1.74)	1.09 (0.71, 1.68)
Tricuspid atresia and stenosis	1	15/1454	1.04 (0.86, 1.27)	1.04 (0.90, 1.21)	1.23 (0.86, 1.75)	2.10 (1.09, 4.06)	1.43 (0.99, 2.06)	1.24 (0.80, 1.93)
Tricuspid atresia and stenosis	2	15/1454	1.03 (0.84, 1.27)	1.03 (0.88, 1.21)	1.19 (0.83, 1.71)	2.13 (1.10, 4.13)	1.40 (0.96, 2.04)	1.20 (0.76, 1.87)
Pulmonary valve stenosis	1	13/1878	1.02 (0.84, 1.23)	1.01 (0.84, 1.20)	0.88 (0.58, 1.33)	1.01 (0.59, 1.74)	1.08 (0.75, 1.55)	1.19 (0.79, 1.78)
Pulmonary valve stenosis	2	13/1878	1.01 (0.83, 1.23)	1.00 (0.83, 1.20)	0.87 (0.57, 1.31)	1.02 (0.59, 1.75)	1.06 (0.73, 1.52)	1.16 (0.77, 1.75)
Coarctation of aorta	1	29/2151	0.99 (0.75, 1.29)	1.00 (0.82, 1.22)	0.99 (0.66, 1.47)	1.54 (0.85, 2.78)	1.26 (0.89, 1.80)	0.97 (0.63, 1.49)
Coarctation of aorta	2	29/2151	0.98 (0.75, 1.28)	1.00 (0.83, 1.21)	0.98 (0.66, 1.46)	1.52 (0.83, 2.78)	1.28 (0.88, 1.84)	0.98 (0.64, 1.51)
Respiratory	1	90/2725	0.94 (0.78, 1.14)	0.93 (0.78, 1.11)	1.02 (0.82, 1.28)	1.00 (0.73, 1.38)	0.98 (0.79, 1.22)	0.93 (0.73, 1.20)
Respiratory	2	90/2725	0.95 (0.80, 1.14)	0.95 (0.80, 1.12)	1.04 (0.83, 1.30)	0.98 (0.71, 1.36)	1.01 (0.81, 1.26)	0.96 (0.75, 1.24)
Oro-facial clefts	1	107/2725	1.01 (0.90, 1.14)	0.99 (0.87, 1.11)	0.94 (0.77, 1.17)	0.95 (0.71, 1.27)	1.00 (0.82, 1.22)	1.07 (0.86, 1.33)
Oro-facial clefts	2	107/2725	1.02 (0.92, 1.14)	1.00 (0.89, 1.12)	0.96 (0.78, 1.19)	0.94 (0.70, 1.25)	1.03 (0.84, 1.25)	1.11 (0.89, 1.39)
Digestive system	1	133/2725	1.08 (1.02, 1.14)	1.06 (1.01, 1.11)	1.14 (0.97, 1.35)	1.22 (0.93, 1.60)	1.11 (0.93, 1.32)	1.03 (0.84, 1.26)
Digestive system	2	133/2725	1.09 (1.03, 1.15)	1.07 (1.02, 1.12)	1.18 (1.00, 1.40)	1.20 (0.91, 1.57)	1.15 (0.97, 1.37)	1.09 (0.89, 1.33)
Abdominal wall defects	1	39/2725	1.02 (0.83, 1.25)	1.02 (0.87, 1.20)	1.09 (0.79, 1.49)	2.00 (1.17, 3.41)	1.00 (0.72, 1.39)	0.95 (0.65, 1.38)
Abdominal wall defects	2	39/2725	1.06 (0.89, 1.26)	1.05 (0.92, 1.21)	1.16 (0.84, 1.60)	1.92 (1.13, 3.29)	1.09 (0.80, 1.49)	1.04 (0.71, 1.53)
Urinary	1	336/2725	1.02 (0.96, 1.09)	1.00 (0.95, 1.06)	1.05 (0.93, 1.18)	1.05 (0.88, 1.24)	1.06 (0.95, 1.19)	1.04 (0.92, 1.19)
Urinary	2	336/2725	1.03 (0.97, 1.10)	1.02 (0.96, 1.07)	1.07 (0.95, 1.20)	1.02 (0.85, 1.21)	1.10 (0.98, 1.23)	1.09 (0.95, 1.24)
Hypospadias	1	51/2298	1.02 (0.83, 1.25)	0.98 (0.78, 1.22)	1.15 (0.88, 1.52)	1.74 (1.11, 2.73)	1.00 (0.76, 1.33)	1.08 (0.78, 1.50)
Hypospadias	2	51/2298	1.04 (0.86, 1.25)	1.00 (0.82, 1.22)	1.19 (0.90, 1.57)	1.70 (1.08, 2.68)	1.04 (0.79, 1.38)	1.14 (0.81, 1.58)
Limb	1	210/2725	1.02 (0.94, 1.10)	1.01 (0.95, 1.08)	1.00 (0.87, 1.17)	1.14 (0.91, 1.41)	1.03 (0.89, 1.19)	0.93 (0.79, 1.10)
Limb	2	210/2725	1.04 (0.97, 1.12)	1.04 (0.98, 1.10)	1.05 (0.90, 1.22)	1.10 (0.89, 1.37)	1.09 (0.95, 1.26)	1.01 (0.85, 1.19)

[a]Spatial exposure inter-quartile ranges for all subjects combined: NO_2 (12.2), NO_x (27.1), PM_{10} (3.0), PM_{coarse} (3.6), $PM_{2.5}$ (2.6), $PM_{2.5}$ absorbance (0.73). [b]Model 1:adjusted for maternal age, conception season, year of birth, socio economic index. Model 2: Model 1+ maternal age. [c]The number of controls varies to avoid collinearity when there were no cases in certain years.

Supplemental Material, Table S5. Stratified analyses by year of birth/termination: Models for spatial exposure to air pollutants: adjusted odds ratio (OR)[a] and 95% confidence interval (CI) for each inter-quartile range increase [b] in exposure.

Congenital anomaly	Model[c]	N cases / N controls[d]	NO_2 OR (95% CI)	NO_x OR (95% CI)	PM_{10} OR (95% CI)	PM_{coarse} OR (95% CI)	$PM_{2.5}$ OR (95% CI)	$PM_{2.5}$ absorbance OR (95% CI)
All non-chromosomal	1	970/1278	0.97 (0.92, 1.03)	0.99 (0.94, 1.03)	0.94 (0.86, 1.02)	0.98 (0.87, 1.11)	0.96 (0.88, 1.04)	0.89 (0.81, 0.98)
All non-chromosomal	2	1203/1591	0.98 (0.94, 1.03)	0.98 (0.94, 1.02)	0.97 (0.90, 1.05)	1.03 (0.92, 1.15)	0.94 (0.87, 1.02)	0.95 (0.87, 1.03)
Neural Tube Defects	1	56/1278	0.81 (0.61, 1.06)	0.81 (0.63, 1.06)	1.00 (0.77, 1.32)	0.68 (0.46, 1.00)	0.93 (0.71, 1.22)	0.90 (0.66, 1.22)
Neural Tube Defects	2	83/1591	0.96 (0.81, 1.15)	0.98 (0.85, 1.13)	1.05 (0.83, 1.31)	1.08 (0.77, 1.53)	0.98 (0.78, 1.23)	0.93 (0.72, 1.22)
Congenital heart disease	1	398/1278	0.90 (0.81, 1.01)	0.94 (0.85, 1.02)	0.95 (0.85, 1.07)	0.91 (0.77, 1.08)	1.00 (0.89, 1.11)	0.88 (0.77, 1.00)
Congenital heart disease	2	425/1591	0.99 (0.92, 1.05)	0.98 (0.92, 1.04)	0.96 (0.86, 1.08)	0.91 (0.77, 1.07)	0.95 (0.85, 1.06)	0.93 (0.82, 1.06)
Coarctation of aorta	1	32/1278	1.09 (0.92, 1.29)	1.07 (0.93, 1.24)	1.41 (1.05, 1.88)	1.22 (0.71, 1.09)	1.46 (1.05, 2.03)	1.30 (0.89, 1.89)
Coarctation of aorta	2	37/1591	1.07 (0.99, 1.17)	1.06 (0.99, 1.14)	0.93 (0.65, 1.34)	1.58 (0.93, 2.67)	1.09 (0.80, 1.50)	1.08 (0.75, 1.56)
Abdominal wall defects	1	20/1278	0.96 (0.62, 1.47)	0.99 (0.70, 1.38)	0.92 (0.56, 1.52)	1.46 (0.72, 2.98)	0.93 (0.58, 1.48)	0.76 (0.45, 1.30)
Abdominal wall defects	2	35/1591	1.03 (0.87, 1.22)	1.01 (0.85, 1.19)	1.19 (0.87, 1.63)	1.69 (0.98, 2.93)	0.95 (0.67, 1.34)	1.08 (0.74, 1.59)

[a]Models adjusted for maternal age, conception season, year of birth/termination, socio economic index [b] Spatial exposure inter-quartile ranges for all subjects combined: NO_2 (12.2), NO_x (27.1), PM_{10} (3.0), PM_{coarse} (3.6), $PM_{2.5}$ (2.6), $PM_{2.5}$ absorbance (0.73).[c] Model 1: Year of birth/termination between 1994 and 1999. Model 2: Year of birth/termination between 2000 and 2006. [d] The number of controls varies to avoid collinearity when there were no cases in certain years.
